# Reasons for and Facilitating Factors of Medical Malpractice Complaints. What Can Be Done to Prevent Them?

**DOI:** 10.3390/medicina56060259

**Published:** 2020-05-27

**Authors:** Bianca Hanganu, Magdalena Iorga, Iulia-Diana Muraru, Beatrice Gabriela Ioan

**Affiliations:** 1 Legal-Medicine Department, Faculty of Medicine, “Grigore T. Popa” University of Medicine and Pharmacy of Iasi, 700115 Iasi, Romania; bianca-hanganu@umfiasi.ro (B.H.); beatrice.ioan@umfiasi.ro (B.G.I.); 2 Behavioral Sciences Department, Faculty of Medicine, “Grigore T. Popa” University of Medicine and Pharmacy Iasi, 700115 Iasi, Romania; diana.muraru@umfiasi.ro; 3 Faculty of Psychology and Education Sciences, “Alexandru Ioan Cuza” University of Iasi, 700554 Iasi, Romania

**Keywords:** medical malpractice, doctor–patient relationship, communication, complications, diagnostic error, preventive measures, retrospective study

## Abstract

*Background and objectives.* Medical malpractice is an increasing phenomenon all over the world, and Romania is not spared. This matter is of concern as it has a significant impact on the physicians and the patients involved, as well as on the health care system and society in general. The purpose of our study was to perform an insight analysis on the reasons for medical malpractice complaints as well as the factors that facilitate the complaints to identify specific ways to prevent them and, implicitly, to improve the medical practice. *Materials and Methods*. The authors conducted a retrospective study of the medical malpractice complaints registered in the period 2006–2019 at the Commission for monitoring and professional competence for malpractice cases in the region of Moldova, Romania, collecting data on both the patients and the medical professionals involved. *Results*. The authors analyzed 153 complaints directed against 205 medical professionals and identified 15 categories of reasons for complaints, the most significant being related to the occurrence of complications, and to the doctor–patient interaction (e.g., communication, behavior, informed consent). The most frequently reported medical specialties were obstetrics and gynecology, emergency medicine, general surgery, and orthopedics and traumatology. Emergency medicine was often involved in complaints suggesting an over utilization of this department in our country and the need for health policies, which could divert the large number of patients accessing emergency medicine towards primary care. *Conclusions*. Regarding the dysfunctions in the doctor–patient relationship frequently claimed by patients, the authors concluded that doctors need special undergraduate training and periodic updating during their practice for them to be able to adequately address the challenges of interacting with their patients.

## 1. Introduction

The medical practice has been regulated throughout its entire historical evolution by specific legal and ethical norms, which are aimed at carrying out the medical act in optimal conditions, protecting the patients, and sanctioning the doctors who violate the norms of good practice [1]. Medicine and science, in general, enjoy quasi-constant advancements, which bring multiple benefits through their wide applicability [2]. Nonetheless, by increasing public expectations, emphasizing the publicity of patient rights [1,3] and the marketing of medicine [3], the progress in technology places a greater burden on the shoulders of the doctors, which often leads patients to have unrealistic expectations and causes them to sanction any mistake or any result that does not coincide with their expectations [4]. This, in turn, leads to tensions in the doctor–patient relationship [2]. Thus, the current medical practice risks being dominated by fear of potential malpractice complaints and their consequences on the medical staff.

The increasing number of medical malpractice complaints represents an alarming reality worldwide, with reports from various health services in Europe, the United States of America, and Australia confirming it [5,6]. Thus, Jena et al. (2011) showed that in the United States of America, 7.4% of medical professionals are accused of malpractice each year [7]. Likewise, a 2009 study showed that about 4% of the 108,000 physicians insured by a German insurance company faced a medical malpractice accusation each year [8]. In the United Kingdom, the number of complaints against general practitioners (GP) increased more than two-fold during a period of 5 years, between 2007 and 2012 [6]. In Romania, the malpractice complaints submitted to the court annually increased from 8 in 2008 to 65 in 2017, with a total of 331 in the period 2007–2018, 62 (18.73%) of these being registered in the eight counties included in our study [9].

The increase in the number of malpractice complaints is a burden for both the doctors and the medical system through their impact on the medical practice, as it can lead to the approach to the medical act in a defensive manner, as well as through the judicial procedure which usually lasts for a long time and involves significant costs for physicians and medical institutions [2,6].

Defensive medicine involves requesting more medical tests, more medical opinions, prescribing more drugs, more referrals to specialized examinations, refusing to perform certain high-risk procedures, or even refusing to assist patients with severe illnesses. These practices are not always for the benefit of the patients; on the contrary, they subject them to unnecessary and sometimes risky interventions, increase the costs of medical care, and decrease the level of satisfaction among physicians [2].

To reduce the negative impact of the judicial process for the resolution of malpractice complaints on doctors and patients, an extrajudicial procedure (e.g., no-fault system, mediation) has been introduced in many countries alongside the judiciary system. This extrajudicial procedure has a number of advantages for both the patient and the doctor and the medical system in general. In out-of-court settlements, complaints are resolved more quickly and efficiently, with lower costs compared to judicial resolution. An out-of-court settlement promotes the disclosure of medical practice incidents, which can increase patients’ trust in physicians and can improve the physician–patient relationship, thus being preventive, and increases patient safety by improving the quality of medical care [10,11,12]. Likewise, resolving the complaints out-of-court decreases the level of psychological stress to which the doctor and the patient are subjected, and the doctor can continue or resume his/her professional activity without fear or pressure resulting from the complaint being made in court or released in the press. These latter aspects are particularly important as after the disclosure of the complaints in the press and the presentation in court, even if the doctor is exonerated, the associated psychological stress could irreversibly damage his/her professional life [12].

In Romania, the out-of-court procedure for resolving medical malpractice complaints was legislated in 2006, when the Commissions on monitoring and professional competence for malpractice cases were created. These Commissions carry out their activity at the level of the Public Health Directorates (PHD) in each county and in Bucharest, and their composition is mixed, covering the activity of physicians, dentists, nurses, midwives, and pharmacists. The working procedure of these commissions provides that patients who consider themselves harmed as a result of the medical act, or their family members (in the case of deceased or incompetent patients), may submit complaints accompanied by documents certifying the harm. The complaints are analyzed with the support of medical experts in the targeted medical specialty to determine whether or not the patients have suffered as a result of malpractice. The medical experts who analyze the complaints belong to various medical specialties and are included in a list available nationwide. They are designated randomly, by drawing lots, and their fee is paid by the plaintiff. To prove an act of malpractice, four criteria must be met cumulatively: (a) the existence of a deed produced during a prevention, diagnosis, or treatment activity; (b) the deed is causing patrimonial or non-patrimonial (moral) damage to the patient; (c) the guilt of the medical professional; (d) the causal relationship between the deed and the prejudice [13].

This study aimed to analyze the reasons for the complaints of medical malpractice submitted to the commissions at the PHDs in the region of Moldova, as well as the factors that facilitate the complaints, to identify ways to prevent the complaints and, implicitly, to improve the medical practice. Moldova is a large territory located in the northeast and partially the southeast of Romania, between the Carpathians Mountains and the Prut River, and includes 8 counties out of the total of 41, plus the capital city, Bucharest (Bacau, Botosani, Galati, Iasi, Neamt, Suceava, Vaslui, and Vrancea), with a total surface of about 46,000 km^2^ and around 4 million people.

To our knowledge, this research is the first of its kind in Romania.

This study is part of a broader doctoral research, which aims to identify methods to prevent the complaints of medical malpractice submitted by patients and to reduce their impact on the medical staff.

## 2. Materials and Methods

### 2.1. Data Collection

The authors conducted a retrospective multi-centric study of malpractice complaints registered in the period 2006–2019 at the Commissions on monitoring and professional competence for malpractice cases in the region of Moldova, which includes 8 counties (Bacau, Botosani, Galati, Iasi, Neamt, Suceava, Vaslui, Vrancea). Data were collected from complaints submitted by patients who were treated in either public (*n* = 26) or private (*n* = 14) medical institutions or their family members and from decisions made by the commissions in each case. The collection, storage, and processing of data were done with respect for the confidentiality of personal data of doctors and plaintiffs.

The collected data concerned, on the one hand, the reasons for the complaints and, on the other hand, the persons who made the complaints (patients or family members) and the doctors who were involved in the respective cases. More specifically, the data regarding the persons who filed the complaints concerned the following aspects: the socio-demographic characteristics of the patients (gender, age, residence area); the existence of multiple pathologies; if the patient died or not; the petitioner who filed the complaint (the patient or their family members); the number of days of hospitalization; the existence of multiple hospitalizations. The data about the doctors concerned: medical specialty, professional degree, and gender.

The analysis of the reasons underlying the complaints led to the identification of 15 categories, depending on the central, essential elements mentioned in the complaints. For identifying the categories of reasons, the authors used an inductive approach. All authors read the reasons as stated by the complainants multiple times, and to ensure rigor, they met several times to analyze them. In this way, the authors identified 15 categories related to aspects of the medical act itself or to the doctor–patient relationship and decided together on the inclusion of each reason in one of the established categories.

### 2.2. Ethical Approval

The research was approved by the Research Ethics Commission of the “Grigore T. Popa” University of Medicine and Pharmacy of Iasi, Romania, No. 16434 / July 30, 2019. 

### 2.3. Statistical Analysis

The collected data were analyzed using IBM SPSS Statistics, version 23. Percentages, means, and standard deviations were used for the descriptive analysis. The independent samples *t*-Test was used in the case of variables where there were two independent groups to determine if there were any statistically significant differences between the means of these groups. The Chi-square test was used to test the association between categorical variables.

## 3. Results

We analyzed 153 complaints directed against 205 medical professionals (physicians, nurses, physiotherapists, and dentists). In 12 cases, the plaintiff also complained about the hospital. Of the people who filed complaints, 82 (53.6%) were male, and 71 (46.4%) were female. Their ages ranged from 0 (new-born) to 90 (M = 37.21, SD = 24.38); however, the analyzed documents did not specify the age of the plaintiffs in 12 cases. Close to half of the patients were deceased (43.8%). Most of the plaintiffs live in urban areas (67.3%), and most of them filed the complaints in Iasi (30.1%) and Galati (28.1%) counties. In more than half of the cases, the plaintiffs were members of the patient’s family (in case of deceased or incompetent patients). All the data related to the socio-demographic characteristics of the plaintiffs are presented in Table 1.

The medical specialties in which most complaints were found were (1) obstetrics and gynecology, with 24 (15.7%) claims against 29 physicians (14.14%), (2) emergency medicine, with 18 (11.8%) claims against 20 physicians (9.75%), (3) general surgery with 16 (10.5%) claims against 20 physicians (9.75%), and (4) orthopedics and traumatology with 12 (7.8%) claims involving 19 physicians (9.26%).

We identified 15 categories of reasons for complaints. Categories, examples of specific reasons for each category and the most involved specialties in each category are shown in Table 2.

We tested the association between the reasons mentioned above and some of the study variables concerning the plaintiff (residence area, gender, if the patient was deceased, if the plaintiff was the patient or a family member, if the patient was hospitalized multiple times, if the patient was a new-born) and we found several statistically significant results. Six of the reasons for complaint (treatment errors, reasons suggested by colleagues, inappropriate communication among team members, administrative reasons, legal reasons, and lack of competence/professionalism) were not associated with any of the aforementioned variables. The other nine had associations with the study variables in various ways as showed below.

### 3.1. Reasons Related to Technical Aspects of the Medical Activity

#### 3.1.1. Complications

“Complications” as a reason for complaint was associated with:

- Whether the patient was deceased or not: χ2(1) = 20.913, *p* < 0.001; Phi coefficient = −0.371, *p* < 0.001, indicating a small size effect; more specifically, “complications” was more likely to be a reason for complaint in cases where the patients did not die (54.7%) compared with the cases where they did (18.2%);
- Gender: χ2(1) = 7.929, *p* = 0.005; Phi coefficient = 0.228, *p* = 0.005, indicating a small size effect; more concretely, “complications” was more likely to be a reason for complaint in the cases of female patients (50.7%) compared to male patients (28.4%);
- Whether the plaintiff was the patient or a family member: χ2(1) = 10.402, *p* = 0.001; Phi coefficient = −0.262, *p* = 0.001, indicating a small size effect; in this case, patients were more likely to complain about the occurrence of complications (54.0%) than their relatives (28.1%);
- Whether the patient had multiple hospitalizations: χ2(1) = 4.370, *p* = 0.037; Phi coefficient = 0.174, *p* = 0.037, indicating a small size effect; specifically, patients with multiple hospitalizations were more likely to complain about complications (49.1%) compared to those without multiple hospitalizations (31.8%).

In addition, there was a statistically significant difference in number of hospitalization days between patients who complained about complications and those who did not: t(128) = −4.824, *p* < 0.001; the patients from the former category had a higher mean of hospitalization days (M = 24.85) compared to the latter (M = 8.71).

#### 3.1.2. Negligence

“Negligence” as a reason for complaint was associated with whether the plaintiff was the patient or a family member: χ2(1) = 4.351, *p* = 0.037; Phi coefficient = 0.169, *p* = 0.037, indicating a small size effect. In this case, relatives were more likely to complain about negligence (32.6%) than the patient (17.5%).

There was a statistically significant difference in number of hospitalization days between patients who complained about negligence and those who did not: t(128) = 2.900, *p* = 0.004. Patients who complained about negligence had a higher mean of hospitalization days (M = 8.94) compared to those who did not (M = 17.12).

#### 3.1.3. Diagnostic Errors

“Diagnosis errors” as a reason for complaint was associated with whether the patient had multiple hospitalizations: χ2(1) = 4.094, *p* = 0.043; Phi coefficient = 0.168, *p* = 0.043, indicating a small size effect. Patients with multiple hospitalizations were more likely to complain about diagnosis errors (19.3%) compared to those without multiple hospitalizations (8.0%).

#### 3.1.4. Delay (in Examination, in Diagnosis, in Treatment)

“Delay” as a reason for complaint was associated with:

- Whether the patient was deceased or not: χ2(1) = 13.239, *p* < 0.001; Phi coefficient = 0.295, *p* < 0.001, indicating a small size effect; delay was more likely to be a reason for complaint in cases where the patients died (22.7%) compared to the cases where they did not (3.5%);
- Whether the plaintiff was the patient or a family member: χ2(1) = 5.166, *p* = 0.023; Phi coefficient = 0.184, *p* = 0.023, indicating a small size effect; delay was more likely to be a reason for complaint by family members (16.9%) compared to patients (4.8%).

#### 3.1.5. Lack of Diagnosis

“Lack of diagnosis” as a reason for complaint was associated with gender: χ2(1) = 7.402, *p* = 0.007; Phi coefficient = −0.221, *p* = 0.007, indicating a small size effect. More concretely, lack of diagnosis was more likely to be a reason for complaint in the cases of male patients (9.9%) compared to female patients (0.0%).

### 3.2. Reasons Related to the Relationship between Patients and Medical Staff

#### 3.2.1. Lack of Information/Informing Deficiency

“Lack of information/informing deficiency” as a reason for complaint was associated with:

- Whether the plaintiff was the patient or a family member: χ2(1) = 7.212, *p* = 0.007; Phi coefficient = 0.218, *p* = 0.007, indicating a small size effect; lack of information/informing deficiency was more likely to be a reason for complaint for family members (22.5%) compared to patients (6.3%);
- Whether the patient was deceased or not: χ2(1) = 4.223, *p* = 0.040; Phi coefficient = 0.167, *p* = 0.040, indicating a small size effect; lack of information/informing deficiency was more likely to be a reason for complaint in cases where the patients died (22.7%) compared to those cases in which the patients did not die (10.5%);
- Whether the patient was newborn or not: χ2(1) = 13.857, *p* < 0.001; Phi coefficient = 0.302, *p* < 0.001, indicating a medium size effect; in cases where patients were newborn, lack of information/informing deficiency was more likely to be a reason for complaint (62.5%) compared to those in which the patients were not newborn (13.2%).

#### 3.2.2. Inappropriate Language

“Inappropriate language” as a reason for complaint was associated with whether the patient was newborn or not: χ2(1) = 8.082, *p* = 0.004; Phi coefficient = 0.231, *p* = 0.004, indicating a small size effect. In cases where patients were newborn, inappropriate language was more likely to be a reason for complaint (37.5%) compared to those in which the patients were not newborn (7.6%).

#### 3.2.3. Informed Consent

“Informed consent” as a reason for complaint was associated with whether the patient was newborn or not: χ2(1) = 4.662, *p* = 0.031; Phi coefficient = 0.175, *p* = 0.031, indicating a small size effect. In cases where the patients were newborn, informed consent was more likely to be a reason for complaint (25.0%) compared to those in which the patients were not newborn (5.6%).

#### 3.2.4. Inappropriate Behavior

“Inappropriate behavior” as a reason for complaint was associated with the residence area (rural or urban): χ2(1) = 4.296, *p* = 0.038; Phi coefficient = 0.172, *p* = 0.038, indicating a small effect size. In cases where patients lived in rural areas, inappropriate behavior was more likely to be a reason for complaint (16.7%) compared to those who lived in urban areas (5.8%).

## 4. Discussion

This study allowed the analysis of data gathered during 14 years of activity of the Commissions on monitoring and professional competence for cases of malpractice in the Moldova region of Romania. Our results showed several findings that could be the starting point for formulating prevention methods designed to the reduction in the number of malpractice claims.

Our study showed that the ground for complaints are both reasons related to technical aspects of the medical activity and reasons related to the relationship between patients and medical staff, these results are consistent with literature data [14]. The analysis of these reasons is important so as to improve the medical practice by identifying various prevention methods that target both the medical practice and the doctor–patient relationship, to improve medical services provided to patients, and to protect the medical staff from complaints [2].

In our study we found that over half of the complaints were registered in two counties (Iasi and Galati). The large number of cases in these two counties (compared to the other counties included in the research) is due, most likely, to the fact that Iasi and Galati are university centers, with clinical hospitals, which can provide a higher level of medical services and can treat more severe cases, and where many patients from other counties in Moldova are referred from smaller hospitals. Likewise, many patients request on their own initiative a referral by the general practitioners to specialists working in university centers, sometimes contrary to their recommendation [15].

Literature data show that there are no medical specialties spared by malpractice complaints, but some specialties have a higher risk compared to others [16].

In our study, the most frequently reported medical specialties are obstetrics and gynecology, emergency medicine, general surgery, and orthopedics and traumatology. These results are partly consistent with those of other studies. Obstetrics and gynecology, general surgery, and orthopedics and traumatology also occupy the first places in studies conducted in other countries, such as the United Kingdom, China or the United States of America [4,7,17].

The increased risk of certain medical specialties for malpractice claims is mainly due to the particularities of the medical services provided and the patients treated. Physicians in obstetrics and gynecology, for example, treat not only one patient, but often at least two (mother and child) or even three, if we take into account the concerns of the future father, while by taking care of the reproductive system of the woman, it also deals with potential future patients, the reproduction of the human species, thus becoming a specialty of the entire family [4]. Patients accessing obstetrics and gynecology services often expect for more than what a doctor can actually do, asking for flawless results of the medical interventions, such as safe labor when the child is to be given birth [18]. Becoming a parent can be an overwhelming issue, and the perinatal period is characterized by plenty of emotions related to the health status of the newborn. As such, this emotional burden on the parents may become a trigger for complaints against professionals in this field when the newborn is not healthy [19].

Physicians in orthopedics and traumatology are at high risk because they have to take care of patients’ work capacity [4], which may have significant social and professional implications.

The increased risk for complaints of malpractice in surgical specialties is underlined by Jena et al. (2011), who showed that allegations of medical malpractice occur quite frequently in this specialty. Those authors found that 88% of physicians in at-risk surgical specialties (neurosurgery, thoracic and cardiovascular surgery, general surgery) had one complaint against them until the age of 45, and the number increased to 99% by the age of 65 [7]. Patients who need surgery usually suffer from severe diseases for which they expect doctors to work miracles. As the medical procedures are more complex, the associated risks increase in a directly proportional manner [4].

A peculiar result of our study was the fact that emergency medicine was the second most claimed specialty in terms of the number of complaints, and the third most claimed specialty in terms of the number of doctors involved, which is in contradiction with other studies that found a small number of complaints against the medical personnel who work in emergency medicine units, patients and the general public taking into account that physicians in this specialty work in critical conditions [20]. However, a similar result was reported in the study conducted by Hwang et al. (2018) in Taiwan, where this specialty occupied the third place, with 8.5% of complaints, after obstetrics and orthopedics [18]. Furthermore, unlike various studies published in the literature in which there is an increased number of complaints against general practitioners [21,22,23], in our study family medicine (which in Romania is equivalent to general practice) was involved in only one complaint against doctors from several specialties.

Our finding that the ratio between the number of complaints in Emergency Medicine and that in family medicine was reversed compared to other studies finds its explanation in the fact that Romanian patients use emergency medicine unit services excessively, to the detriment of primary health care services [15,24]. Patients often shunt the family doctors, abusively calling emergency medical services, even in non-life-threatening situations. This overuse of emergency medicine to the detriment of family medicine (which is underused) is also demonstrated by the fact that many ambulance requests, especially during regular working hours, are resolved at the patients’ homes, representing situations that could have been solved by the family doctor [15]. An essential reason for patients requesting excessive consultations from emergency services is the possibility to be seen by a doctor without a referral from the family medicine physician or in case they do not have health insurance. Although health insurance is mandatory for all Romanian citizens, European Commission reports show that in 2017, 11% of the population did not have it [25]. There are certain diseases (e.g., genetic diseases, diabetes, tuberculosis, myasthenia gravis, increased obstetrical risk in pregnant women, peptic ulcer, mental illnesses) [15,26] which can be assessed or followed up by a specialist physician directly in the outpatient department, without a referral from the family doctor [24], which could further explain the lower number of complaints to the latter, by the lower addressability. Moreover, the primary health care system in Romania is not accessible to everyone, about 2.5% of the population do not have access to a family doctor [27].

In our study, 9.2% of the patients complained about the doctors’ inappropriate language, 9.2% complained about their inappropriate behavior, and 15.7% complained about the lack of information or the deficiency in offering information. These issues raise an alarm about how doctors approach and relate to their patients and the fact that deficiencies in the doctor–patient relationship can lead to a malpractice complaint.

Our study highlights that individuals from rural areas were more likely to report inappropriate behavior compared to those from urban areas. In rural areas, the relationship between the doctors and their patients is much closer [28,29]. This closer relationship might result in the patient preferring to wait a long time (sometimes even a few hours) to be examined by their own doctor when the latter is very busy, or even to postpone a consultation if their doctor is not available, instead of contacting another physician [28]. Interpersonal relationships are generally closer in rural areas, where communities are small, people communicate more with each other (for example, they usually all greet even if they do not know each other), they live in communion. The rural environment is characterized by greater social integration, with friendly and neighborly support and involvement by the community, compared to the urban environment [30]. The rural patient, accustomed to close interpersonal relationships in their area of residence, both with the community and with the physician, is disturbed when urban physicians, with less time allocated to patients, interact less with the patient, behave more distantly and sometimes arrogantly, as some patients in our study pointed out. These issues become more relevant when the outcome of the medical intervention is not in line with the patient’s expectations.

Data from the literature show that in addition to the actual harm caused to the patient, as an independent reason, an important role in formulating the complaint has a series of triggers, especially related to the relationship between doctor and patient [31], to how the patient is approached by the doctor [5], and to the fact that the doctor often leaves the patient without explanations as to the reasons for the failure of the medical act [32]. At the same time, there are studies showing that despite the occurrence of harm to the patient, the proper way in which physicians have related to their patients contributed to the patients’ decision to not complain about the medical act [33].

The ability of inter-human relationships, in the form of communication and the attitude of the doctor towards the patient [5], strongly influences the medical practice. Communication failure often predisposes the occurrence of adverse events [34]. Moreover, patients are more likely to complain about the doctor after the occurrence of an adverse event if the doctor–patient relationship is dysfunctional [3]. Thus, the study conducted by Veerman et al. (2019) showed that at least 10% of patients were disappointed in how their doctors behaved and communicated with them, in the sense that their problems were not given due consideration and that their doctors were too busy to discuss with them [5].

A study conducted in the United States of America that analyzed the malpractice complaints in general surgery showed that 34% of complaints were related to poor communication [35].

Doctor–patient communication triggers complaints of malpractice also from the perspective of obtaining the medical history. Failure to obtain an accurate medical history predisposes to misdiagnosis and treatment errors, which generates patient dissatisfaction and subsequent malpractice complaint [36]. On the other hand, a good communication may prevent the patient from making a malpractice complaint even if the result of the medical act is not as expected, because the level of communication between doctor and patient allows the latter to understand the situation and accept the result [37]. Regarding the starting point of conflicts, both family members and physicians recognize the implications of poor communication [38]. Doctors who disregard their patients’ feelings and concerns, who provide little information, who do not have the patience to listen to or are not open with their patients are more likely to be reported, compared to their colleagues who communicate more efficiently with patients [3].

Our study showed that the lack or deficiency in communication was more likely to be reported by the family than by the patient, rather when the patient had died and when the patient was a newborn. These elements suggest the need of the family to be informed in difficult moments, such as the unexpected death of a family member or the health condition of their children. Moreover, when death occurs in a child, the need for the parents to receive information and explanations is even higher, given that usually a child is not supposed to die from natural causes [20]. Communicating bad news can be a difficult task for doctors, with a great emotional load on both sides. To provide it in an appropriate way, the doctors need specific training and protocols [39]. In general, the perinatal period is associated with a strong emotional load [19], and as the reality of a newborn’s health problem adds significant a degree of vulnerability [40], it can strongly upset the new parents, some of them perhaps experiencing parenthood for the first time. Therefore, the need for realistic, accurate information about the medical situation becomes essential [38,40]. Moreover, it is necessary that the doctor–parent interaction be grounded on clear, prompt, and compassionate communication by doctors [38,40].

In our study, inadequate informed consent or lack thereof was reported in 6.5% of cases. This result is closely related to the results of other studies, such as one conducted in Australia, which showed that 5% of negligence claims and conciliated complaints were related to the process of obtaining the informed consent [32]. Obtaining the informed consent is an important part of the process of communicating with the patient. Agarwal et al. (2018) identified aspects related to professionalism and inadequate informed consent as factors favoring the initiation of malpractice complaints in cases involving spine surgery [41].

Our results showed a higher probability of complaining about the lack of consent in cases where patients were newborns. This result was related to another result of our study indicating that the lack or deficiency of communication was a more common reason for complaint by the patient’s family (when the patient died, or the patient was a newborn or incompetent). Data from the literature indicate the need for a family-centered approach in the medical settings for the care of newborns, requiring the adequate approach of the parents [40,42,43]. In a family-centered approach, the parents and the doctor form a partnership in which the parents are offered the opportunity to actively participate in the care of their child [42,43]. Sarin and Maria (2019) reported that parents often show distress, frustration, and alienation when they are not involved in caring for their own child [42], thus creating the premises for complaints when the evolution of the case is not favorable.

Patients who complain about their doctors for the damage they suffered as a result of a misdiagnosis or treatment error [2] may want to obtain compensation, to find out what happened [3], to get an explanation, or they may want the doctor to admit their mistake [3,31] and express their regret [31]. Patients may also want to prevent the occurrence of similar incidents in the future or for justice to be done, i.e., those responsible for the mistake to be held accountable [3,44].

Our results showed that in 59 (38.5%) cases the reason for complaints was the occurrence of a complication of the medical act, the complaints being directed towards 75 (36.58%) physicians. The most frequently claimed specialties for this reason were obstetrics and gynecology (15 complaints, 19 doctors), general surgery (6 complaints, 10 doctors) and orthopedics and traumatology (7 complaints, 9 doctors), all of them major surgical specialties, which, by their nature, involve an intrinsic risk of the occurrence of additional harm during surgical interventions which are often complex [4]. Some of the complications are related to the complexity of the disease, but others are related to errors that could have been prevented [45]. Our results show that women were more likely to claim complications than men and for the complaint to be made by the patients rather than their relatives, probably because the patient is the one who endures the physical suffering associated with the occurrence of a complication and the subsequent necessary treatments. The complaint for the occurrence of a complication of the medical act was associated with a greater number of hospitalization days and with multiple hospitalizations because the occurrence of a complication requires additional medical or surgical treatment.

The diagnostic error was a reason for complaint identified in 19 (12.4%) cases, involving a number of 28 (13.65%) individual physicians, the three most involved specializations for this reason being pediatrics (4 complaints, 6 doctors), obstetrics and gynecology (2 complaints, 3 doctors), and orthopedics and traumatology (2 complaints, 5 doctors). This was a more likely reason for complaint for patients with multiple hospitalizations. The lack of diagnosis was a reason for complaint in 8 (5.22%) cases, involving 11 (5.36%) doctors, the most involved specialty for this reason being general surgery (2 complaints, 4 physicians). Although the literature data most often refer to diagnosis errors in general (lack of diagnosis, misdiagnosis, and delay in diagnosis), the results of our study showed a lower percentage compared to those reported in other studies. For example, 32.1% complaints related to diagnosis were reported by Gupta et al. (2018), 38.8% of them concerning hospitalized patients [46]. Gupta et al. (2018) observed a decrease in this percentage during the 13 years of the study period (January 1, 1999-December 31, 2011), an aspect that can be explained by the increasing accessibility of the diagnosis techniques, especially in the medical imaging field [46]. The diagnostic errors in outpatients were estimated at 5.1% of the cases [46]. Schaffer et al. (2017) showed that diagnostic errors were the most reported reason in cases of paid claims, being found in 31.8% of these cases [47]. Diagnostic errors were the most common cause of complaint in the study performed by Saber Tehrani et al. (2013), which showed that diagnostic errors were accompanied by the highest costs and the highest degree of danger for patients [48].

Misdiagnosis is often associated with mistreatment and may require multiple re-admissions in the hospital, increasing morbidity and the associated costs [46]. Agarwal et al. (2018) found 31.6% claims for delayed diagnosis and 32.7% claims for failure to provide appropriate treatment, which may underline that acute patients need prompt and appropriate care [41].

In our study the lack of diagnosis was reported by men rather than women, a similar result being obtained by Gupta et al. (2018) [46]. According to the empathizing-systemizing theory, proposed by Baron-Cohen and cited by Zaidi (2010), men tend to systematize, seek solutions, understand, and build different systems, going to the root of the problems, so that everything becomes clear to them. Therefore, male patients are dissatisfied by the lack of a clear explanation for their health problems and start their own search for solutions or answers [49].

The consequences of diagnosis errors have been analyzed by several studies. For example, Zwaan et al. (2010) identified a 6.5% death rate in a hospital due to adverse events [50]. Gupta et al. (2018) showed that diagnosis errors result in 47.4% of deaths and 33.9% of disabilities [46]. In our study, diagnosis errors were not significantly associated with the patient’s death, this being reported only in 10.6% (*n* = 7) of the deceased patients. A reason for complaint strongly associated with the patient’s death in our study was the delay of the medical act. The most involved specialties for claiming delay in the medical act were general surgery (4 complaints, 5 physicians) and emergency medicine (4 complaints, 4 physicians). Delay as a reason for medical malpractice claim in emergency medicine is again associated with the overutilization of the emergency services where the waiting time to receive medical care is inversely proportional to the severity of the emergency.

In cases when the patient is deceased or incompetent (i.e., by age, by physical or psychical disability), the complaint is submitted by a family member. In our study, this occurred in more than half of the cases (i.e., 58.8%). This aspect has multiple facets. Except for the already mentioned reason regarding the stress and emotions surrounding the perinatal period, the parents of an injured child might be preoccupied by the future of their offspring in case of disability [19]. Likewise, the death of a loved one may cause a great deal of distress, with material or moral prejudice, the family thus being entitled to ask for explanation and compensation in case of the physician’s malpractice leading to the misfortunate event [1]

### Strengths and Limitations

This study has several strengths and limitations. Regarding the strengths, this study is the first of its kind to be conducted in Romania and covered a significant geographic part of the country, Moldova being one of the largest Romanian regions. Therefore, the results offer an image of the situation for about 20% of the country’s entire population and can represent the starting point for studies in the other regions, to have a national perspective on this subject. Second, the research analyzed complaints registered for 14 years, offering an objective data analysis. Third, the results are discussed and explained in opposition with other medical systems showing the most vulnerable medical specialties in Romania compared to other countries.

The limitation of the study stems from the fact that some of the cases had missing data regarding the socio-demographic characteristics of the plaintiffs and some of the claims did not contain precise information on the number of doctors involved. This limitation was the result of the fact that the patients gave a personal account of the events, instead of having access to a default form with specific items for each category of information.

## 5. Conclusions

Complaints of medical malpractice continue to follow an ascending trend worldwide, and their consequences are multifaceted, affecting both the medical staff and patients and society in general. Therefore, it becomes significantly necessary to identify solutions meant to reduce the number of complaints, a goal that could be met by conducting studies aimed at formulating recommendations in this regard.

The results of our study showed that the most frequently involved specialties were obstetrics and gynecology, emergency medicine, general surgery, and orthopedics and traumatology. Particularly in this list was the presence of the emergency medicine, a result explained by the overuse of these services in our country and the underutilization of primary health care services. Therefore, health policies are needed to divert the large number of patients accessing emergency medicine to primary care.

Many of the aspects the plaintiffs complained about were represented by deficiencies in the interaction between doctor and patient or his/her family (lack of or deficiency in information, the doctor’s inappropriate behavior or language, failure to obtain informed consent). Based on this finding, we consider that doctors need special training during their undergraduate medical studies, as well as periodic updating during their career to meet the challenges of communication and relationships with patients and their families.

## Figures and Tables

**Table 1:** Socio-demographic characteristics of the plaintiffs.

Variables		N (%)
Gender	Male	82 (53.6)
	Female	71 (46.4)
County	Iasi	46 (30.1)
	Vaslui	9 (5.9)
	Botosani	13 (8.5)
	Suceava	16 (10.5)
	Galati	43 (28.1)
	Neamt	8 (5.2)
	Bacau	8 (5.2)
	Vrancea	10 (6.5)
Deceased	Yes	67 (43.8)
Residence area	Rural	42 (27.5)
	Urban	103 (67.3)
	Missing data	8 (5.2)
Plaintiff	Patient	63 (41.2)
	Patient’s family	90 (58.8)
Multiple	Yes	57 (37.3)
hospitalizations		
	Missing	8 (5.2)
New-born	Yes	8 (5.2)
Age (M ± SD)		37.21 (±24.38)
Hospitalization days (M ± SD)		14.86 (±18.86)

**Table 2:** Categories of reasons.

Category	Examples of Reasons Mentioned by Patients and Family Members in Proxy Position	No of Cases	No of Physicians	Most Involved Specialties *
**I. Reasons Related to Technical Aspects of the Medical Activity**				
Complications	Injuries produced while admitted to the hospital and during surgical intervention Infection of the surgical incision Post-surgery complication Retained foreign object	59 38.6%	75 36.58%	OG GS OT
Treatment errors	Total hysterectomy instead of myomectomy Wrong maneuver leading to dislocation of the prosthesis Wrong epidural injection	45 29.4%	66 32.19%	OG Dentist GS
Negligence	Patient left unsupervised Superficial clinical examination Overlooking important findings Failure to recommend/refer for additional examinations Failure to hospitalize the patient	40 26.1%	45 21.95%	EM
Diagnostic errors	Small cell carcinoma vs. large cell carcinoma Establishing diagnosis without performing all the necessary tests Intestinal obstruction vs. botulism	19 12.4%	28 13.65%	Pediatrics OT OG
Delay	In diagnosis In transferring the patient In examining the patient (waiting too long in the waiting room) In performing investigation In child delivery In treatment	18 11.8%	20 9.75%	GS EM Pediatrics
Lack of competence/ Professionalism	Incompetence Lack of knowledge Insufficient medical knowledge	13 8.5%	19 9.26%	OG EM Pediatrics
Lack of diagnosis	Missed the bone fissure on the X-ray Missed the injury of the left ear Missed the nuchal cord Missed the rupture of the aortic aneurism	8 5.2%	11 5.36%	GS OT Pediatrics
Deficiencies in filling out the medical file	Failure to mention all the information in the medical file Mentioning more diagnoses than the patient actually had Replacing the medical file, etc.	7 4.6%	8 3.90%	OG GS
Administrative	Improper functioning of the emergency system	3 2.0%	4 1.95%	OT
**II. Reasons Related to the Relationship between Patients and Medical Staff**				
Lack of information/informing deficiency	Failure to mention the risks Failure to offer explanations Failure to provide information about the diagnosis	24 15.7%	30 14.63%	GS Pediatrics OG EM
Inappropriate behavior of the physician	Insufficient interaction Expecting monetary incentive Arrogance Distant attitude Disrespect	14 9.2%	18 8.78%	EM PS OH
Inappropriate language	Allusion to ethnicity Inappropriate tone of voice Offensive words	14 9.2%	19 9.26%	OG EM Pediatrics
Suggestion made by other doctors	Blaming the late transfer on the previous doctor Blaming the patient’s previous doctor for brutal procedures Blaming the patient’s previous doctor for errors in treatment in open discussions with fellow colleagues Inciting the patient to legal proceeding against previous doctors and medical units	14 9.2%	13 6.34%	GS EM Ophthalmology
Failure in obtaining informed consent	Apical resection without obtaining the patient’s consent Removing the ovarian adherences detected during the surgery to prepare the ovaries for IVF, against patient’s expressed refusal of IVF Failure to follow the previously established therapeutic plan and proceeding to change without prior informing the patient	10 6.5%	18 8.78%	OG Dentist
Inappropriate communication among medical team members	Failure to inform fellow doctors about the special technique he was performing/about the surgical intervention Lack of communication between physicians who were treating the patient	3 2.0%	5 2.43%	Ophthalmology

* OG—obstetrics and gynecology, OT—orthopedics and traumatology, GC—general surgery, EM—emergency medicine, PS—plastic surgery, OH—oncology and hematology.
